# Improving performance of Zambia Defence Force antiretroviral therapy providers: evaluation of a standards-based approach

**DOI:** 10.9745/GHSP-D-13-00053

**Published:** 2013-08-14

**Authors:** Young Mi Kim, Joseph Banda, Webby Kanjipite, Supriya Sarkar, Eva Bazant, Cyndi Hiner, Maya Tholandi, Stephanie Reinhardt, Panganani Dalisani Njobvu, Adrienne Kols, Bruno Benavides

**Affiliations:** a Jhpiego/USA, an affiliate of Johns Hopkins University, Baltimore, MD, USA; b Jhpiego/Zambia, an affiliate of Johns Hopkins University, Lusaka, Zambia; c Zambia Defence Force (ZDF), Defence Force Medical Services (DFMS), Lusaka, Zambia

## Abstract

**Background:**

The Zambia Defence Force (ZDF) has applied the Standards-Based Management and Recognition (SBM-R®) approach, which uses detailed performance standards, at some health facilities to improve HIV-related services offered to military personnel and surrounding civilian communities. This study examines the effectiveness of the SBM-R approach in improving facility readiness and provider performance at ZDF facilities.

**Methods:**

We collected data on facility readiness and provider performance before and after the 2010–2012 intervention at 4 intervention sites selected for their relatively poor performance and 4 comparison sites. Assessors observed whether each facility met 16 readiness standards and whether providers met 9 performance standards during consultations with 354 returning antiretroviral therapy (ART) clients. We then calculated the percentages of criteria achieved for each readiness and performance standard and conducted bivariate and multivariate analyses of provider performance data.

**Results:**

Facilities' ART readiness scores exceeded 80% before the intervention at both intervention and comparison sites. At endline, scores improved on 4 facility readiness standards in the intervention group but on only 1 standard in the comparison group. Multivariate analysis found that the overall provider performance score increased significantly in the intervention group (from 58% to 84%; *P*<.01) but not in the comparison group (from 62% to 70%). The before-and-after improvement in scores was significantly greater among intervention sites than among comparison sites for 2 standards—initial assessment of the client's condition and nutrition counseling.

**Conclusion:**

The standards-based approach, which involved intensive and mutually reinforcing intervention activities, showed modest improvements in some aspects of providers' performance during ART consultations. Further research is needed to determine whether improvements in provider performance affect client outcomes such as adherence to ART.

## Abstract (precis)

A detailed standards-based performance approach modestly improved providers' performance and facility readiness to offer antiretroviral therapy. The approach included mutually reinforcing activities: (1) training, (2) supportive supervision, (3) assessments of service quality, and (4) facility-based action plans.

## BACKGROUND

The incidence of HIV infection in Zambia has declined by more than one-fifth since 2000.^1^ Still, the prevalence of infection among adults remains high—an estimated 12.5% in 2011.^2^ Zambia has rapidly scaled up HIV counseling, testing, treatment, and care over the past 10 years. Government policy provides for free antiretroviral therapy (ART) for anyone with a CD4+ cell count below 350/mm^3^. Estimates of ART coverage vary from 72% to 90% of the more than 420,000 adults in need of treatment in 2011.^1^^,^^3^ However, rising demand for ART and other HIV-related services has stressed the capacity of available infrastructure, drug supplies, trained staff, and management and support systems.^4^ Improvements in service delivery are also needed to ensure that ART is started early and to promote adherence to lifelong treatment, which is crucial to maximizing the prevention and treatment benefits of antiretroviral (ARVs) drugs and minimizing the development of drug resistance.^3^

The need for more and better ART services holds true for military as well as civilian populations in Zambia. The demographic makeup of the military and conditions of deployment have led to higher HIV prevalence in the armed forces than in general populations across sub-Saharan Africa.^5^-^6^ In Zambia, a 2004 seroprevalence study found that HIV prevalence among Zambia Defence Force (ZDF) personnel was 29%,^7^ compared with a national prevalence of 16% at that time.^8^ The ZDF established an HIV/AIDS program focused on prevention in 1993 and since has steadily expanded HIV-related services.^9^ These efforts have had a positive impact on a range of indicators. For example, the proportion of ZDF personnel ever-tested for HIV rose from just 18% in 2004 to 84% in 2011,^10^ compared with 23% of the general population who had been voluntarily tested for HIV and received results as of 2009.^11^ The number of ZDF health facilities offering comprehensive and integrated HIV/AIDS services increased from just 5 in 2006 to 28 in 2013, with a concomitant rise in the number of clients served.

More recently, the ZDF has made the quality of HIV-related services a priority.^7^ To that end, the ZDF began introducing Jhpiego's Standards-Based Management and Recognition (SBM-R®) approach at some hospitals and clinics in 2006. This quality improvement initiative has the potential to influence care beyond the military population because ZDF's 54 facilities serve surrounding communities as well as military personnel and their families: civilians make up four-fifths of the clients seen at ZDF facilities. The military health system accounts for 16% of health services in Zambia.^12^ Thus, successful interventions at ZDF facilities can serve as models for the Ministry of Health (MOH) system, with which it is deeply integrated.

Successful interventions at ZDF facilities can serve as models for the MOH system.

### The SBM-R Approach: 4 Steps

The SBM-R approach to quality improvement uses a set of detailed standards to guide health care workers and measure progress in service delivery.^13^ It looks not only at provider performance, but also at the functioning of management, drug procurement, and other systems.

**Figure 1.**
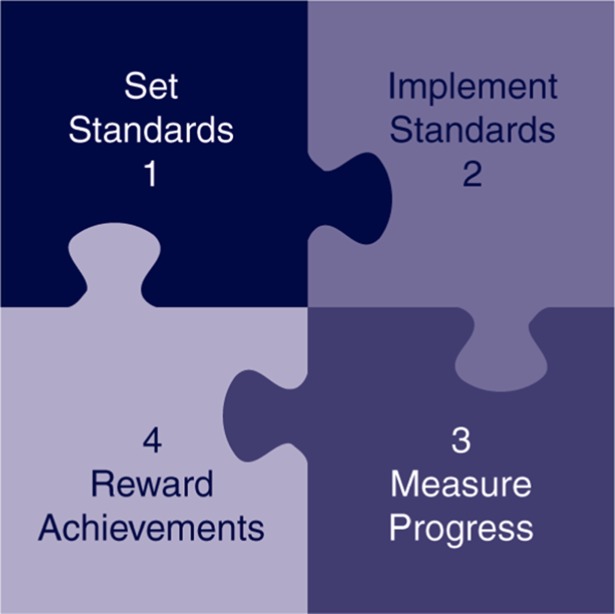
The 4 Steps of the Standards-Based Management and Recognition (SBM-R) Approach

1. The first of 4 steps in the SBM-R process (Figure 1) is to establish evidence-based and locally relevant standards that define the desired level of performance in a service delivery area. Each performance standard is divided into a series of specific tasks, known as verification criteria for purposes of assessment. In Zambia, for example, the performance standard for assessing ART clients for adverse reactions includes 6 verification criteria: inquiring about sleeping problems, inquiring about nausea and vomiting, inquiring about yellow eyes, inquiring about shortness of breath, offering reassurance, and treating side effects appropriately. Because SBM-R performance standards are so detailed, they function as job aids that help individual providers improve their performance. Primarily, however, they serve as assessment tools that enable facilities to measure compliance with accepted standards of care.
2. The second step in SBM-R is to implement the performance standards at each facility, under the direction of a team of managers and providers. With outside support, the facility team conducts a baseline assessment of services, using a mix of direct observations, structured interviews, and record review to assess whether each verification criterion is met. Team members follow a step-wise process that leads to an action plan (see box). They take a relatively simple approach to analyzing each gap identified, using root cause analysis, and look within the facility for realistic solutions that can be implemented with existing personnel and resources. Only if that fails does the team seek outside assistance, which in Zambia meant going up the chain of command to the unit commander. It takes about 1 week for the facility team to conduct the baseline assessment, analyze the findings, and create an action plan. Implementing different portions of the action plan can take a few months to a year, depending on how complicated they are and whether outside assistance is needed.
3. The third step in SBM-R is to monitor the facility's progress toward meeting the standards by periodically repeating the performance assessment and working to address remaining performance gaps.
4. When sufficient progress is made, the facility reaches the fourth and final step in the SBM-R process: recognizing and rewarding achievements.

**Box. Using SBM-R to Develop Action Plans**

SBM-R team members:

- Analyze assessment findings ↓
- Identify weaknesses in service delivery ↓
- Determine the causes ↓
- Develop low-cost, local solutions ↓
- Implement an action plan to address performance gaps

Although SBM-R shares some elements with other approaches to quality improvement, SBM-R is designed to have certain advantages in low-resource settings:^14^

- It focuses more on practical solutions and less on analysis.
- It helps transfer learning because assessment tools can be used as job aids and for self-monitoring.
- It achieves quick and consistent results.
- It motivates health workers, because they actively participate in the process as members of the facility team overseeing SBM-R.
- It keeps costs low and is easier to scale up because it requires little additional manpower, mobilizes existing resources, and focuses on low-cost solutions.

Measuring the impact of quality improvement interventions on health care has proved difficult. This may explain the lack of rigorous studies, despite the many projects that have tested quality improvement interventions in developing countries. Recent reviews of the literature have concluded that the evidence base for quality improvement remains weak, although some studies suggest a positive impact.^15^-^16^ Rawlins and colleagues found that the SBM-R approach improved the performance of reproductive, maternal, and child health services in Malawi.^17^ However, SBM-R has not been widely applied to HIV treatment, and there are no evaluations of its effectiveness for ART services. Nor has there been an assessment of the suitability of the approach for military health systems. SBM-R may be more challenging in a military setting because of its collaborative team approach, which requires facility staff at every level to work together to identify and address performance gaps. This style conflicts with that of the military's hierarchical command structure.

## METHODS

### Study Purposes

This study evaluates the effectiveness of the SBM-R approach in improving ART services in a military setting. We examine 2 components of quality. The first, facility readiness, assesses whether adequate infrastructure, supplies and equipment, and management and support systems are in place to support good-quality services. The second, provider performance, assesses whether providers meet standards for good-quality care during their interactions with returning ART clients.

### Study Design and Sample

This study employed a quasi-experimental design that collected data at 2 points in time from intervention and comparison sites. Baseline data were collected from August 10 through October 21, 2010, taking 1 to 4 days per facility. The intervention launched on September 15, 2010 and continued up to and beyond the second round of data collection. At 2 intervention sites, we collected endline data from March 13 through April 13, 2012, after the SBM-R intervention had been in place for 18 to 19 months. Because of an administrative transition, we had to collect endline data earlier at the other 2 intervention sites and the 4 comparison sites; these data were collected from November 29 through December 8, 2011. At that point, the SBM-R intervention had been in place for 15 months.

Of the ZDF's 54 facilities, 16 had already implemented SBM-R and so were excluded from the study. The ZDF selected 4 of the remaining 38 facilities as intervention sites, based on their sufficient caseload and need for improvement. We selected 4 non-intervention sites for comparison; they were matched as closely as possible with intervention sites on ZDF branch, number of beds, and size of catchment population. It proved impossible to match sites based on ART service volume. Also, we did not match comparison sites with intervention sites on performance or perceived need for improvement. The 8 health facilities in the sample represent all 3 branches of the ZDF: they include 2 Zambian Air Force facilities, 2 Zambian National Service facilities, and 4 Zambian Army facilities.

We invited all health care providers responsible for delivering ART services to participate in the study, and all of them agreed. A total of 21 providers were interviewed at baseline and 28 at endline—1 to 5 providers at each facility in each round. Due to problems assigning unique ID numbers to providers during data collection, we cannot be certain whether the same or different individuals were interviewed in each round of data collection, nor can we link providers with the consultations observed. We took steps in the statistical analysis to account for potential non-independence in the outcome measures.

Clients who had already started ART and were returning for a follow-up visit were eligible to participate in the study. Providers asked clients' permission to have their consultations observed. Assessors observed every client who came for an ART follow-up consultation with a participating provider during their facility visits. The same procedure was followed at intervention and comparison sites. The goal was to observe 25 consultations at each facility, but that was not always possible. The number of clients observed per facility during each round ranged from 21 to 27, with 3 exceptions (9, 13, and 16 observations). In the end the procedure yielded a convenience sample of 354 clients who were returning for ART follow-up visits—81 clients at the baseline and 81 clients at the endline at intervention sites and 94 clients at the baseline and 98 clients at the endline at the comparison sites. It is unlikely that the same clients were observed during both rounds of data collection, but we do not know for certain. We did not ask clients whether they were military personnel, but it is reasonable to assume that the majority were civilians, given that civilians make up four-fifths of all clients seen at ZDF facilities. Service data suggest that the clients observed represent about one-fifth of the facilities' ART client population.

### The Intervention

The SBM-R intervention at ZDF sites was designed to improve 2 service areas: ART and the prevention of mother-to-child transmission (PMTCT) of HIV. While this article focuses on the impact on ART services, simultaneous efforts were proceeding at intervention sites to improve PMTCT services. We are analyzing and presenting those findings separately.

The MOH, Jhpiego, ZDF, and the University Teaching Hospital adapted, for Zambia, the ART standards developed in South Africa; they were based on international best practices as summarized in World Health Organization (WHO) guidelines. The standards were first implemented in 2006 at the start of the larger SBM-R project in Zambia. They were further refined to fit the Zambian context in 2010, which resulted in shorter tools that were easier to implement.

Next, 2 staff members from each intervention site attended a 3-day workshop on SBM-R, after which ZDF and Jhpiego staff visited the facilities to launch the intervention. During a 3-day visit to each facility, they oriented managers to SBM-R, introduced service providers to the tools and desired outcomes, and coached providers. A team of 4 to 7 staff members were assembled at each facility to lead the SBM-R process for both ART and PMTCT. Each team included the facility in-charge and 3 to 6 service providers who played diverse roles at the facility; they included clinical officers, nurses, medical assistants, and pharmacy technicians. Teams' membership changed over the course of the study, as some individuals were transferred to other ZDF facilities or left for temporary deployments. Colleagues and supervisors oriented their replacements to SBM-R. However, ART providers at intervention sites did not change: all of them remained in place from baseline to endline. We do not know whether the comparison sites experienced any provider turnover.

During the initial site visit, ZDF and Jhpiego staff worked with the facility team to conduct a formal assessment of ART and PMTCT services, using the SBM-R tools to identify strengths and weaknesses in provider performance and support systems. They systematically examined conditions at the facility, observed interactions with clients, and recorded whether or not each verification criterion was met. Afterwards, the team reviewed the results and used a “Why Tree” approach to determine the causes of each problem: they asked why a problem occurred and then turned the answer into another “why” question. They repeated this process until satisfied that they had found the root cause of the problem.

### The Action Plan

Following the site visit review, the team developed a detailed action plan to improve service quality. The plan listed each problem, along with a solution, the person responsible, and an expected date of completion. The SBM-R teams and managers at all of the facilities considered implementing the action plan to be the most important part of SBM-R and worked at it daily. Some action points were handled entirely at the facility level: for example, to facilitate collaboration, a manager compiled a list of local organizations providing health services. Sometimes outside assistance was needed: for example, a team found that the poor treatment of Military Medical Assistants (MMAs) contributed to inadequate staffing. MMAs are recruited from the ranks of the ZDF to fill shortages in the number of health workers; they are initially trained to provide basic services, such as bandaging wounds and taking vital signs, but may eventually gain a skill set equivalent to that of an enrolled nurse. Officers viewed MMAs as casual workers and frequently ordered them to perform non-health-related tasks. The SBM-R teams raised the issue with unit commanders and ZDF headquarters, who fully committed the MMAs to health care.

Shortly after and in response to the baseline SBM-R assessments, providers received 5 days of onsite ART training to strengthen knowledge of the standards and improve clinical skills. The training was competency-based and included lectures, role plays, and opportunities to practice with clients. ZDF and Jhpiego also provided needed supplies and equipment to facilities, such as gloves and blood pressure cuffs.

To encourage facility teams to continue pursuing problems, ZDF and Jhpiego arranged 3-day supportive supervision visits to each facility approximately twice a year. During these visits, a team of 2 or 3 supervisors observed consultations and mentored staff. Supervisors came mostly from the ZDF's largest referral hospital and had extensive experience with SBM-R assessments. Supervisors coached individual providers on meeting ART standards. Also, they met with the facility team to review and, if necessary, revise the SBM-R action plan. None of the teams at the intervention sites took the initiative to repeat the SBM-R performance assessments by themselves during the course of the study. Instead, they focused on implementing the action plan and measured their progress by how many action points they had resolved. The study did not last long enough for any of the sites to reach the last step in the SBM-R cycle, recognition and rewards.

### Data Collection

We collected data from 2 sources: observations of the facility's readiness to offer ART services and observations of ART follow-up consultations. The units of analysis are the facility and the individual consultation, respectively. The baseline and the endline employed the same data collection tools (see supplementary materials).

*Facility Readiness Observations:* At each site, assessors completed a facility observation instrument based on SBM-R tools. It covered operations that directly support ART services, such as the supply of antiretroviral drugs, and general support systems that are essential to delivering good-quality health care services of all kinds, such as adequate staffing. As shown in Table 1, the instrument covered 8 standards for ART readiness and 8 standards for general readiness; together they included 95 verification criteria. Assessors marked each one as observed or not observed for the facility as a whole.

**Table 1.** Facility Readiness Standards, Standards-Based Management and Recognition

ART Readiness Standards	No. of Verification Criteria	General Readiness Standards	No. of Verification Criteria
ART drug requisition: system for reordering drugs is properly managed	5	Staffing: sufficient staff are available for daily operations	3
ART drug storage: drugs are properly stored, tracked, and issued	9	Infrastructure: staff and client comfort and safety are assured	6
Pharmacist counseling: information on ART drugs is offered to clients	9	Supplies: sufficient stocks of critical supplies are available	3
Individual monitoring plan: plan is developed with client to monitor adherence and toxicities	8	Management systems: referral, scheduling, communication, and evaluation systems are working	7
Checking adherence: follow-up visits reinforce adherence to treatment, answer questions, and dispense drugs	11	Waste disposal: waste is handled and disposed of properly	5
Access to lab tests: clients' access to required laboratory tests is ensured	2	Client records: client files are kept confidential and are readily available	5
Blood drawing: infection prevention and other guidelines are followed	9	Health information system: timely collection, analysis, and reporting	5
Transport of blood samples: proper collection and transport to laboratory	3	Performance improvement: ongoing implementation of performance improvement activities	5
**Maximum possible ART facility readiness score**	**56**	**Maximum possible general facility readiness score**	**39**

Abbreviation: ART, antiretroviral therapy.

*Observation of Consultations:* Before the consultation, assessors asked returning clients about their age, education, number of prior ART visits, and duration of therapy. During the consultation, assessors completed an observation checklist that included 9 performance standards for ART follow-up consultations (Table 2). Assessors noted whether the provider performed each of 48 verification criteria.

**Table 2.** Performance Standards for Provision of ART Services, Standards-Based Management and Recognition

Standard	No. of Verification Criteria	Content
Initial assessment of patient's condition	6	Greetings, registration, ask about patient well-being, review medical history
Assessment of opportunistic infections	3	Rule out pneumocystis pneumonia, cryptococcal meningitis, and tuberculosis
Assessment of adverse reactions	6	Inquire about sleeping problems, nausea, yellow eyes, shortness of breath, etc., and offer reassurance
Assessment of potential drug interactions	3	Ask about new medications, document concurrent medications, check for drug interactions
General health assessment	3	Inquire about contraception, pregnancy, alcohol and recreational drug use, depression; perform targeted physical exam; request and review laboratory tests
Verification of how patient is taking ART and cotrimoxazole	9	Check medication schedule, supplies, missed doses; reinforce adherence; address patient concerns
Addressing identified issues, as needed	8	Manage infections, adverse reactions, laboratory abnormalities; make referrals for social services
Concluding the consultation	5	Address patient questions, plan return visit, complete registers and applicable forms
Nutrition counseling	5	Discuss diet, food preparation, boiling drinking water, hand washing
**Maximum possible ART follow-up score**	**48**	

Abbreviation: ART, antiretroviral therapy.

### Training of Data Collectors

To ensure the quality of the data, we recruited assessors who had experience with field work and trained them on the purpose of this study, the data collection tools, recruitment procedures, consent process, data collection, and ethical issues. Two assessors were hired to collect the data for each round of data collection. All 4 were physicians with considerable experience in ART services. They were third-party staff from the MOH who did not work at the ZDF facilities being assessed. ZDF personnel assisted only in helping the assessors gain admission to the military sites.

### Ethical Considerations

The University of Zambia Biomedical Research Ethics Committee and the Johns Hopkins Bloomberg School of Public Health Institutional Review Board approved this study. Observations and interviews took place in private, and providers and clients gave their informed consent.

### Data Analysis

For facility readiness, we calculated the percentage of verification criteria achieved for each readiness standard and overall scores for ART readiness and general readiness (Table 1). For provider performance, we calculated the percentage of verification criteria achieved for each performance standard as well as an overall score for provider's performance during ART follow-up consultations (Table 2).

We conducted both bivariate and multivariate analyses on provider performance data. (Small sample sizes did not permit further analysis of data collected on facility readiness.) The initial bivariate analysis calculates the gain or decline in percentage achieved scores from baseline to endline separately for the intervention and comparison groups. We used a t-test to detect whether the change from baseline to endline within each group was statistically significant. Further, a multivariate analysis estimates the effect of time on performance outcomes by intervention group status. In the multivariate models, the outcome variable is the number of achieved or performed verification criteria in a standard. Generalized linear regression (GLM) models with Poisson distribution and log link function were used. The GLM model with Poisson distribution was selected after comparing different models, including negative binomial regression, using the Akaike information criterion. The total number of verification criteria is included in the model as an offset term. In addition to adjusting for ZDF branch, the independent variables included the time point (baseline or endline), the evaluation group (intervention or comparison), and the interaction of these terms. The interaction term compares the change in percentage achieved scores (both magnitude and direction) from baseline to endline between intervention and comparison groups. The models also included a cluster-adjusted robust variance estimator, as data obtained within 1 facility are correlated.^18^ All analyses were performed using Stata 12.0 (College Station, TX).

## RESULTS

### Provider Characteristics

A total of 21 ART service providers were interviewed at baseline (12 in the intervention group and 9 in the comparison group), and 28 were interviewed at endline (14 each in the intervention and comparison groups). About half were nurses or nurse-midwives (47.6% at baseline and 53.6% at endline). The rest were clinical officers (42.9% at baseline and 28.6% at endline) and Medical Military Assistants (MMAs) (9.5% at baseline and 14.3% at endline). There was no significant difference between comparison and intervention sites in the distribution of provider types or in providers' age, sex, experience in the ZDF, or years at the facility.

On average, ART providers were 36.0 years old (standard deviation [SD] = 7.9) at baseline and 35.5 years old (SD = 6.6) at endline. Over half were male (57.1% at baseline and 67.9% at endline). On average, they had served as a ZDF health care provider for around a decade (mean 9.8 years, SD = 8.7, at baseline, and mean 10.1 years, SD = 6.7, at endline). On average, ART providers had worked at the same facility for 4.6 years (SD = 5.80) at baseline and 6.1 years (SD = 4.81) at endline. At baseline, 88.9% of providers in the intervention group and 91.7% in the comparison group reported receiving training on ART in the preceding year (*P* = .83). Since training was part of the intervention, at endline all providers in the intervention group reported recent training on ART.

### Client Characteristics

All clients observed were returning ART clients. At baseline, most of the clients observed were male (Table 3). At endline, however, the comparison group was mostly female, while the intervention group remained mostly male; the difference was significant (*P*<.01). The mean age of ART clients was in the mid-30s. Over 80% of ART clients had completed at least primary schooling. About 11% of female ART clients at baseline were pregnant, compared with about 8% at endline.

**Table 3.** Percentage Distribution of ART Client Characteristics in Zambia Defence Force Facilities, by Round of Data Collection and Study Group

	Baseline			Endline		
Characteristics	Comparison Group (n = 94)	Intervention Group (n = 81)	*P* value	Comparison Group (n = 98)	Intervention Group (n = 81)	*P* value
**Sex**						
Male	60.9	50.6	.18^a^	32.7	53.1	.006^a^
Female	39.1	49.4		67.4	46.9	
**Age, in years**						
Mean (SD)	37.3 (8.3)	33.0 (9.4)	.001^b^	34.3 (11.9)	35.8 (10.8)	.39^b^
**Educational attainment**						
Some primary	12.1	6.3	.14^a^	19.0	17.3	.01^a^
Primary or	52.8	45.0		57.9	39.5	
some secondary						
Secondary	35.2	48.8		23.2	43.2	
or higher education						
**Pregnant**(among women only)						
Yes	10.8	10.8	.99^a^	7.9	8.3	.94^a^
No	89.2	89.2		92.1	91.7	
**First or later ART follow-up visit**						
First	8.8	1.2	.03^a^	7.0	10.0	.78^a^
Second	3.3	8.6		10.5	10.0	
Third or more	87.9	90.1		82.6	80.0	
**Duration of ART**						
≤1 year	42.7	38.5	.85^a^	36.6	33.8	.63^a^
2 years	40.7	43.6		28.0	23.8	
3+ years	16.5	18.0		35.5	42.5	

Abbreviations: ART, antiretroviral therapy; SD, standard deviation.

*P* values ≤ .05 were considered statistically significant.

a *P* value from χ^2^ test

b *P* value from t-test

Over four-fifths of all clients observed were making at least their third ART follow-up visit. At baseline, a significantly higher proportion of ART clients in the comparison group than the intervention group were making their first ART follow-up visit (8.8% versus 1.2%; *P*<.05). There were no significant differences at endline. Most clients had received ART for at least 2 years. The proportion that had received ART for 3 years or longer increased from 17% at baseline to 36% at endline in the comparison group and from 18% to 43% in the intervention group, reflecting the scale up of ART over time.

### Facility Readiness

*ART readiness scores* were relatively high at baseline, exceeding 80% in both comparison and intervention groups (Table 4). The ART readiness score declined slightly in the intervention group, but this was primarily because of a sharp drop on a single standard, pharmacy counseling, which fell by 18 percentage points. Scores improved on 4 of 8 standards, and the 2 standards with the lowest baseline scores showed gains of 25 percentage points: checking adherence with treatment at follow-up and proper collection and transport of blood samples to the laboratory. There was no room for improvement on another 2 standards, which had scored 100% at the baseline.

Facilities’ ART readiness scores were relatively high at the baseline, exceeding 80% in both comparison and intervention groups.

**Table 4.** Facility Readiness to Offer Good-Quality Services: Percentage of Verification Criteria Achieved, by Data Collection Round and Study Group, Among Zambia Defence Force Health Facilities

Readiness Standards^a^ (No. of Criteria)	Comparison Group (n = 4)			Intervention Group (n = 4)		
	Baseline	Endline	Change (% points)	Baseline	Endline	Change (% points)
**ART facility readiness**						
ART drug requisition (5)	100.0	100.0	0	100.0	100.0	0
ART drug storage (9)	92.6	85.2	−7.4	85.2	91.7	+6.5
Pharmacist counseling (8)	85.1	63.2	−19.9	92.6	75.0	−17.6
Individual monitoring plans (8)	81.3	65.2	−16.1	75.0	78.1	+3.1
Checking adherence (12)	63.2	73.2	+10.0	51.5	76.7	+25.2
Access to lab tests (2)	75.0	66.7	−8.3	100.0	100.0	0
Blood drawing (9)	96.4	77.8	−18.6	100.0	94.4	−5.6
Transport of blood samples (3)	50.0	50.0	0	62.5	87.5	+25.0
*Total ART readiness score (56)*	*83.7*	*78.2*	*−5.5*	*88.5*	*86.2*	*−2.3*
**General facility readiness**						
Staffing (3)	91.7	100.0	+8.3	100.0	91.7	−8.3
Infrastructure (6)	75.0	79.2	+4.2	86.7	95.8	+9.1
Supplies (3)	83.3	88.9	+5.6	100.0	100.0	0
Management systems (7)	100.0	96.4	−3.6	95.8	100.0	+4.2
Waste disposal (5)	80.0	73.8	−6.2	95.0	90.0	+5.0
Client records (5)	95.0	100.0	+5.0	95.0	85.0	−10.0
Health information system (5)	95.0	80.0	−15.0	95.0	100.0	+5.0
Performance improvement (5)	80.0	85.0	+5.0	90.0	100.0	+10.0
*Total general readiness score (39)*	*85.8*	*93.9*	*+8.1*	*92.5*	*95.1*	*+3.4*

Abbreviation: ART, antiretroviral therapy.

The number of criteria observed at each of the 4 facilities was summed for each readiness standard. This sum was calculated as a percentage of the total number of criteria for each standard, multiplied by 4 to include all 4 facilities.

a Missing values removed from numerator and denominator. N/A values recoded as missing.

In the comparison group, the overall ART readiness score declined from 84% at baseline to 78% at endline. Only 1 standard (checking adherence with treatment) showed improvement, while performance declined on 5 other standards and remained constant on 2. The declines exceeded 15 percentage points for 3 standards: ART counseling by pharmacists, individual monitoring plans, and blood drawing.

*General readiness scores* in both the comparison and intervention groups were relatively high at baseline, exceeding 85%, and increased slightly by endline. For most standards, the change was limited. In the intervention group, however, scores rose 10 percentage points for performance improvement and fell 10 percentage points for client records. In the comparison group, the score for the health information system dropped by 15 percentage points.

### Provider Performance

**Figure 2.**
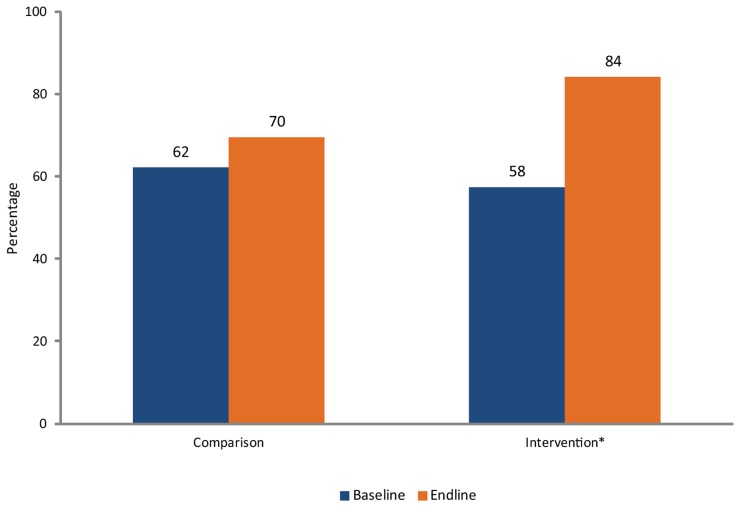
Provider Performance: Percentage of Verification Criteria Achieved n  =  175 at baseline; 179 at endline. *P<.001

At baseline, provider performance was comparable in the 2 study groups (overall ART scores of 62% at the comparison sites and 58% at the intervention sites). During the study, however, the performance of providers during ART follow-up consultations improved more in the intervention group than the comparison group (Figure 2). The overall ART score rose from 58% to 84% in the intervention group. This was a significant increase in both the bivariate (*P*<.001) and multivariate analyses (*P*<.01). In the comparison groups, the overall ART score increased, but not as much (from 62% to 70%). This change was significant in the bivariate analysis (*P*<.001) but not the multivariate analysis.

Some intervention facilities made more progress than others. Three sites had similar overall ART scores at baseline, ranging from 51.3% to 53.4%, and improved to 80.6% to 97.8% at endline. The fourth site had a higher baseline score (68.9%) but showed no progress on the overall ART score (69.5%), although there were marked gains on certain standards.

Scores on 8 of 9 ART performance standards increased significantly in the intervention group, according to the bivariate analysis (Table 5). (The score for concluding the consultation was already at 100% at baseline.) Gains on 6 standards remained significant in the multivariate analysis. Gains exceeded 40 percentage points for 3 standards: assessment of potential drug interactions, addressing identified issues as needed, and nutrition counseling. These standards were among those with the lowest baseline scores.

Gains exceeded 40 percentage points for 3 performance standards that had some of the lowest baseline scores.

**Table 5.** ART Performance: Results of Bivariate and Multivariate Analyses of Percentage of ART Verification Criteria Achieved, by Data Collection Round and Study Condition, Among Zambia Defence Force Health Facilities

	Bivariate Analysis					
ART Performance Standard and Study Condition	% Achieved		Change From Baseline to Endline Within Group		Multivariate Analysis^a^	
	Baseline (n = 175)	Endline (n = 179)	% Points	*P* value	Adjusted *P* value for Change Within Group	*P* value for Interaction
**Initial assessment of patient's condition**						
Comparison	90.1	92.3	+2.2	.08	.67	.001
Intervention	79.6	99.1	+19.5	.001	.001	
**Check for signs of opportunistic infections**						
Comparison	86.2	67.8	−18.4	.001	.28	.13
Intervention	65.2	82.9	+17.7	.001	.33	
**Assessment of adverse reactions**						
Comparison	61.2	50.0	−11.2	.005	.001	.10
Intervention	44.0	78.9	+34.9	.001	.22	
**Assessment of potential drug interactions**						
Comparison	44.2	79.8	+35.6	.001	.001	.26
Intervention	33.1	80.7	+47.6	.001	.001	
**General health assessment**						
Comparison	68.3	83.5	+15.2	.001	.25	.29
Intervention	62.3	93.0	+30.7	.001	.001	
**Verify how patient is taking ART**						
Comparison	57.0	84.6	+27.6	.001	.05	.93
Intervention	59.8	91.0	+31.2	.001	.02	
**Addressing identified issues**						
Comparison	32.9	60.1	+27.2	.001	.20	.93
Intervention	45.0	85.4	+40.4	.001	.001	
**Concluding the consultation**						
Comparison	100.0	98.7	−1.3	.06	.17	.83
Intervention	100.0	98.1	−1.9	.03	.25	
**Nutrition counseling**						
Comparison	4.6	2.0	−2.6	.09	.41	.001
Intervention	1.6	42.3	+40.7	.001	.001	
**Overall ART score**						
Comparison	62.2	69.6	+7.4	.001	.13	.09
Intervention	57.5	84.2	+26.7	.001	.008	

Abbreviation: ART, antiretroviral therapy.

*P* values ≤ .05 were considered statistically significant.

a Results from generalized linear regression with Poisson distribution adjusted for Zambia Defence Force branch and clustering within a facility.

In contrast, in the comparison group performance improved significantly on only 4 standards, according to the bivariate analysis, and fell significantly on 2 standards. In the multivariate analysis 2 gains (verification of how the patient takes ART and assessing potential drug interactions) and 1 decline (assessment of adverse reactions) remained significant. Gains in the comparison group were consistently smaller than those in the intervention group.

The interaction term in the multivariate analysis was significant for 2 standards: the initial assessment of patient's condition (*P*<.001) and nutrition counseling (*P*<.001). This indicates that there was a statistically significant difference in the amount of change over time experienced in the comparison and intervention groups. The interaction term was not significant for the other 7 performance standards or the overall ART score.

## DISCUSSION

### Effectiveness of Intervention

The intervention addressed 2 components of quality: facility readiness and provider performance. SBM-R had a positive, but limited, impact on facility readiness. ZDF facilities were already well-prepared to offer ART services at baseline, in part because of a capacity-building initiative that has worked aggressively since 2007 to strengthen logistics management and the health supply system at all 54 ZDF facilities.^19^ During the course of the SBM-R intervention, however, intervention sites made greater progress on certain readiness standards, such as transport of blood samples, than comparison sites. This may be due to the emphasis that SBM-R teams placed on readiness and management issues in their action plans.

The findings suggest that the SBM-R intervention likely also contributed tqo an improvement in provider performance. Over the course of the intervention, facilities implementing SBM-R experienced substantial gains on every ART performance standard that had room for improvement. In contrast, comparison sites experienced smaller gains on fewer standards, and performance on some standards declined significantly. Notably, there was great improvement at intervention sites in many areas prioritized by the WHO's Global Health Sector Strategy on HIV/AIDS,^20^ including nutrition and co-infections and co-morbidities. Not all intervention facilities experienced the same gains. Informal feedback from program managers suggests that variations in leadership ability, management skills, and understanding of SBM-R among facility managers and SBM-R team members are the primary reasons for the variation in effects.

Not all intervention facilities experienced the same gains. Managers said that this was due to differences in leadership, management, and understanding of SBM-R.

### Implementing SBM-R

The largest expenditures associated with SBM-R were for facility action plans to improve services, notably the purchase of supplies and equipment and ART training. In contrast, costs for SBM-R training were modest because of the onsite group training model used. Assessing the cost of supervision is difficult, because it cuts across multiple practice areas. Providers found that they needed to spend more time with clients to meet SBM-R standards, which could eventually drive up costs as ART caseloads increase. ART follow-up visits with a counselor and a clinician averaged 20 minutes if clients were not experiencing problems and longer if providers had to address an opportunistic infection or other issue.

SBM-R proved to be effective in a military setting despite fears that low-ranking providers would not feel comfortable playing an active role on facility teams. In practice, they viewed themselves as health professionals and acted accordingly. The command-driven nature of the military actually benefited from the intervention, as high-ranking officers took a personal interest in its success. The vocal support of the commanding officer at each site was instrumental in encouraging lower-ranking health workers to embrace quality improvement efforts.

Can lessons learned in a military setting apply to the broader population? In Zambia the military and civilian health systems are closely coordinated. ZDF facilities serve a largely civilian clientele and rely on the MOH for ART guidelines, in-service training workshops, and district supervision teams. Many of the lessons learned from the SBM-R initiative at ZDF facilities can be and are being readily applied to improve the quality of civilian health care. In fact, MOH facilities began introducing SBM-R in 2012. The intervention may work well in settings where the facility in-charge and other authorities give vocal support to it, and where leaders encourage all providers to embrace the quality improvement efforts.

### Study Strengths and Limitations

The study has 2 key strengths. First is the quasi-experimental design, with baseline and endline measures as well as intervention and comparison sites. This design protects against several threats to validity, including history effects (external events that may affect outcomes), maturation (natural improvements over time due to experience), and testing effects (earlier measurements affecting later measurements).^21^

Second, the study relied on direct observations of actual performance conducted by experienced health professionals without ties to the ZDF, using detailed and comprehensive tools that reflect international best practices for low-resource settings and Zambian service guidelines. This approach offers a more objective and reliable assessment of provider performance than interviews, self-reports, simulations, chart reviews, or role plays, which may be biased or reflect idealized situations.^22^

However, interpretation of the findings is subject to certain limitations:

- Participating facilities and individuals were not randomly assigned to the intervention and comparison arms of the study; this is difficult to accomplish in practice.^21^
- Intervention facilities were deliberately selected for SBM-R because they were considered to be in greater need of quality improvement. Therefore, the facility sample may not be representative of all ZDF facilities. The results also are not generalizable to facilities that are not associated with the ZDF.
- The small sample size limited the power of the analysis to identify significant differences between intervention and comparison groups.
- Because providers try harder when under observation (the Hawthorne effect), observations likely overstate providers' usual performance on the job.
- Although inter-observer reliability was checked during training, it was not assessed during the study.
- Sixteen ZDF facilities had already implemented SBM-R prior to this study. It is possible that some of their providers, who were trained on SBM-R, transferred into comparison sites before or during this study. This would tend to narrow differences between the intervention and comparison groups. However, there was no transfer of ART providers out of intervention sites during the study.
- There was a significant difference in client distribution by sex between the intervention and comparison groups at endline.

### Direction for Further Research

This study assessed the short-term impact of the intervention: endline data were collected 15 to 18 months after SBM-R was launched. This was not enough time for intervention facilities to reach the recognition and rewards stage of SBM-R, which has the potential to enhance and sustain impact on provider performance and retention.^13^ Nor was this long enough to assess the sustainability of the SBM-R process. Additional research is needed to assess the impact of the complete SBM-R intervention over longer time periods.

With the scale up of ART across Africa, follow-up services for ART clients are becoming an increasingly important part of the continuum of care for HIV.^23^ While a complex web of personal, social, and structural factors influences clients' adherence to ART regimens, studies in Zambia^24^-^25^ and elsewhere^26^ suggest that quality of care and dissatisfaction with and distrust of health services can play an important role. Quality improvement evaluations need to move beyond provider performance to encompass outcomes such as client perceptions, adherence, and service utilization. This will require collecting both service data and client interview data. Understanding the perspectives of the providers is also important as they are key actors in quality improvement efforts.

## CONCLUSION

ZDF facilities serve an increasing number of clients who are on ART for the long term. A quality improvement initiative that included multiple reinforcing activities—provider training, supportive supervision, detailed performance standards, repeated assessments of service quality, and facility action plans—showed modest improvements in provider performance during consultations with returning ART clients. ZDF has been rolling out SBM-R to 4 facilities a year, and the MOH also has begun to introduce the approach at its facilities, which suggests that the intervention is replicable in both military and civilian settings.

ZDF has been rolling out SBM-R to 4 facilities a year, and the MOH has also begun to introduce the approach at its facilities.
